# Pattern, knowledge and practices of HbA_1C_ testing among diabetic patients in a Kenyan tertiary referral hospital

**DOI:** 10.1186/1744-8603-9-55

**Published:** 2013-11-05

**Authors:** Duncan Mwangangi Matheka, Jeremiah Munguti Kilonzo, Cecilia Muvenyi Munguti, Peter Waweru Mwangi

**Affiliations:** 1 Department of Medical Physiology, School of Medicine, University of Nairobi, P.O. Box 30197-00100, Nairobi, Kenya; 2 Young Professionals Chronic Disease Network, Nairobi, Kenya; 3 Department of Human Anatomy, School of Medicine, University of Nairobi, Nairobi, Kenya

**Keywords:** HbA_1C_, Glycemic monitoring, Availability, Knowledge awareness and practices (KAP), Diabetes mellitus (DM), NCDs, Kenya, Sub-Saharan Africa

## Abstract

**Background:**

Glycated haemoglobin (HbA_1C_) measurement is the currently accepted gold standard biochemical indicator of long-term glycemic control in diabetic patients. The level of knowledge as well as the frequency of use of this test among diabetic patients in Kenya is unknown. The current study aimed to document this among patients attending the diabetes clinic at a national referral hospital in Kenya.

**Methods:**

One hundred and ninety eight diabetic patients (59 male and 139 female) attending the outpatient diabetes clinic at the Kenyatta National Hospital were interviewed on their level of knowledge and use of the HbA_1C_ test, as well as their last HbA_1C_ level. The respondent answers were tabulated, analyzed and summarized. The sample mean, standard deviation and percentages were calculated.

**Results:**

Of the 198 patients interviewed, 11 (5.6%) had type I diabetes mellitus (DM) while 187 (94.4%) had type II DM. One hundred and thirty four patients (67.7%) had heard of the HbA_1C_ test while 64 patients (32.3%) had never heard of the test. Forty patients (20.2%) had at one point done the test while 158 (79.8%) had never done the test. The mean HbA_1C_ level of the 40 patients who had at any one time done the test was 8.5 ± 1.7%, with more than 90% having HbA_1C_ > 8%.

**Conclusion:**

Using self-reported accounts, the current study indicates inadequate knowledge and infrequent testing of HbA_1C_ among diabetic outpatients in Kenya. This lack of knowledge and awareness may lead to increased susceptibility to the development of diabetic complications, and potentially higher healthcare costs among these patients. It is our recommendation that policy makers focus on strategies that address HbA_1C_ test accessibility in Kenya, including financial coverage by the national insurance to access the test in public facilities, so as to effectively monitor and combat DM.

## Background

Diabetes mellitus (DM) is a chronic debilitating non-communicable disease (NCD). In 2011, 366 million individuals had DM, a number that is projected to rise to 552 million by 2030, unless preventive interventions are put in place [1]. Approximately 80% of the burden of DM is in the low-and middle-income countries, and it is growing [1]. In one such country (Kenya), the prevalence of DM has risen from 3.3% in 2007 to 4.2% in 2009, with prevalence rates of up to 10% in some regions [1,2].

The HbA_1C_ test is an indicator of average blood glucose concentrations over the preceding three months. It is a well-established biomarker of long-term glucose control and was approved by the World Health Organization (WHO) for the diagnosis of DM and monitoring glycemic control in people with diabetes [3,4]. Higher HbA_1C_ levels (recommended levels should be maintained at <7%) are associated with the development of diabetic complications, and such an association is not apparent with usual blood glucose tests [5,6].

Despite its value, the HbA_1C_ test is not widely available in Kenya due to its high cost. Although HbA_1C_ test is one of the recommended tests in the Kenyan national clinical guidelines [7], most hospitals have no HbA_1C_ guidelines in place. Moreover, it is not offered in most rural facilities. For these reasons, the required testing of 2 to 4 times a year may be impossible for most diabetic patients in Kenya; hence, there is a need to assess patients’ knowledge and frequency of its use [8].

Health literacy among diabetic patients has been associated with better glycemic control, optimal medication and enhanced individual participation in diabetes self-care [9,10]. Heisler et al. [9] reported that respondents who knew their HbA_1C_ values had better understanding of diabetes care and assessment of their glycemic control than those who did not. Moreover, the lack of awareness may hinder health promotion strategies currently being implemented in Kenya, including screening and media campaigns that assume minimal awareness of such tests.

The current study therefore aimed to establish the state of awareness of the HbA_1C_ test as well as its use among a subset of patients attending the outpatient diabetes clinic at the Kenyatta National Hospital (KNH), a major referral and teaching hospital in Kenya.

## Methods

A cross-sectional descriptive study was conducted between October and November 2012 at the outpatient diabetes clinic of KNH. The hospital has a daily outpatient diabetes clinic with a daily turnover of approximately 20-30 patients, except on Fridays, when there is a major diabetes clinic with a larger turnover. Data were collected on three days of each week (Monday, Tuesday, and Thursday) and patients present on the three days were randomly selected to participate in the study.

One hundred and ninety eight adult type I and II diabetic patients (59 male and 139 female) agreed to participate in the study (response rate: 100%). All participants attended regular follow-up sessions at the diabetes outpatient clinic over the study’s two-month period; and were randomly interviewed before their clinic visit by one of the 3 interviewers (the authors) using a researcher-filled questionnaire. Caution was also taken to ensure that a respondent was not interviewed more than once over the two-month study period. Ethical approval was obtained from the Kenyatta National Hospital-Ethics and Research Committee (KNH-ERC/UA/66). The study protocol was explained to each of the patients, and consenting participants were asked to sign a consent form prior to their interview.

Information was gathered on the patients’ demographic details, level of literacy, type of DM, duration and control of their DM. The patients were also interviewed on their level of knowledge and their practices as appertained to the HbA_1C_ test, as well as their last HbA_1C_ level. This was through a series of short and simple self-reported questions that included: “1. Have you ever heard about HbA_1C_? (Yes, No, Unknown) 2. Have you ever done an HbA_1C_ test? (Yes, No, Unknown) 3. What was your last HbA_1C_ level?” No previous article known to the authors had utilized such an assessment or questions. The respondent answers offered after the above key questions were tabulated, analyzed and summarized using SPSS version 16. The sample mean, standard deviation and percentages were calculated.

## Results

The age of the participants ranged from 20 to 82 years with a mean age of 53.1 ± 0.92 years. Fifty-two patients (26.3%) lived in rural Kenya, while 146 (73.7%) resided in urban areas. Twenty-seven patients (13.6%) had a tertiary level of education, 79 (39.9%) had attained secondary level of education, and 80 (40.4%) had schooled up to primary school level, while 12 (6.1%) had no formal education. The duration of DM ranged from 6 months to 30 years, with 50% of the patients having less than 6 years since diagnosis of their DM. Twenty-five percent of the patients had DM for more than 11 years. Of the patients interviewed, 11 (5.6%) had type I DM, while 187 (94.4%) had type II DM. However, of these patients, only 115 (58.1%) were aware of the type of DM they had been diagnosed with. Of these 115 patients, 11 (9.6%) had type I DM, while 104 (90.4%) had type II DM.

Of all the patients, 134 (67.7%) had heard of the HbA_1C_ test while 64 patients (32.3%) had never heard of the test. Forty patients (20.2%) had at one point done the test while 158 (79.8%) had never done the test. All 40 patients who had done the test were type II DM patients, with 20 (50%) being male and 20 (50%) female. These 40 patients had a mean age of 55.8 years, and an average of 7.7 years after DM diagnosis. The overall mean HbA_1C_ among those who had done the test was 8.5 ± 1.7%, with more than 90% having HbA_1C_ > 8%.

## Discussion

We performed the first study of the frequency and knowledge of HbA_1C_ testing among diabetic patients in Kenya. Using a sample of diabetes patients at the KNH, we found that only 20% of patients had ever done at least one HbA_1C_ check. Among those who did the test, the average HbA_1C_ level was high (more than 90% had HbA_1C_ > 8%). Moreover, we found that only 67.7% of patients interviewed had heard of the HbA_1C_ test. Checks were few and HbA_1C_ knowledge low in both type I and type II diabetic patients.

HbA_1C_ quarterly measurement combined with daily home blood glucose monitoring forms the gold standard of glycemic control in clinical practice [4]. However, HbA_1C_ monitoring is easier said than done, especially in developing countries like Kenya, due to cost constraints [3,4,11].

Currently, HbA_1C_ testing by outpatients in Kenya is paid for by the patient and is not covered by the national insurance. Published reports indicate that the average cost of an HbA_1C_ test in Kenya is US$10 in public health facilities and up to US$25 in private facilities [3]. This cost is prohibitive in a country where 50% of the residents live on less than US$1 a day [11,12] and also contributes to its low availability [4]. The absence of a proper national health insurance plan in Kenya also makes HbA_1C_ financially burdensome. This could explain why a vast majority of our study subjects (80%) had never had the test done. These results are comparable to those of a similar study conducted in Nigeria [13].

Increasing awareness of the test is important. Our study found that a relatively high percentage of diabetes patients had never heard of the test (>30%). One reason for this may be that outpatient HbA_1C_ testing is not part of the package of essential medicines and diagnostics covered by the government through national insurance, and thus is not advertised as being available. This may also be a reflection of the lack of awareness of its utility, as well as the availability of the test among the caregivers coming into contact with the patient. Previous studies have shown that caregivers are the primary sources of information about diabetes to their patients [14].

The relatively low level of awareness of the test as well the low frequencies of testing are noteworthy, particularly since the patient cohort was obtained from a major teaching and referral hospital where one would ordinarily expect cutting edge therapeutic and disease management approaches to be adopted as a matter of course. Further, this is an indication that the situation in lower level health facilities may actually be worse than what is documented in this study. There is a need to carry out studies to document the level of awareness, skill and attitudes among Kenyan health professionals on the HbA_1C_ test in lower level health care facilities and assess existence of relevant training programs initiated to remedy any identified knowledge gaps. Furthermore, a widespread rollout of subsidized or free HbA_1C_ testing in all government health facilities would ensure integration of this test in diabetes management regimens. This is so as to ensure the optimal glycemic control in all patients and hence prevent the development of diabetic complications.

The reported high HbA_1C_ levels (8.5 ± 1.7%), among the 40 patients who had undergone testing in our study are indicative of poor glycemic control that often leads to the development of diabetic complications [8,15]. In these 40 patients with information on their HbA_1C_ level, over 90% had poor glycemic control (HbA_1C_ > 7%). This suggests that poor glycemic control may also be a problem among those not tested. This may be a reflection of the use of treatment regimens or dosages of sub-optimal efficacy and/or poor drug compliance by the patients. It may also reflect a misconception among caregivers that this test should only be used in patients with more serious disease instead of being used in routine management as recommended.

Lastly, besides concerns on the reliability of self-reported data by the participants, results from this study suggest that a small proportion of diabetic patients at KNH have received health education regarding HbA_1C_. There is also a possibility that HbA_1C_ tests may have been done without the knowledge of the patients, and that their results were not discussed with the doctor. Increased patient involvement and proper communication on the part of health care providers, as well as discussion to ensure that the patients understand their management, is critical. With HbA_1C_ approval for glycemic monitoring, health care providers need to educate their patients on its role too [10]. Our future research in this area will focus on patient follow up and outcomes with appropriate HbA_1C_ testing. Moreover, we will focus on studying the patterns of HbA_1C_ testing in each type of DM, since it can be noticed that most patients interviewed in the current study (94.4%) had type II DM.

## Conclusion

The results of the current study indicate inadequate knowledge and monitoring of blood glucose control among diabetic patients in Kenya using the HbA_1C_ test. This signals a lack of access to care and low public awareness about the test and may lead to increased susceptibility to the development of diabetic complications among these patients. The consequences include increasing healthcare costs in a population facing a double burden of disease. It is our recommendation that policy makers focus on strategies that address HbA_1C_ test accessibility in Kenya, including financial coverage by the national insurance to access the test in public facilities, so as to effectively monitor and combat DM. We also call for streamlining of the healthcare infrastructure and national health insurance towards coverage of outpatient clinic costs.

## Abbreviations

DM: Diabetes mellitus; ERC: Ethics Research Committee; HbA1C: Glycated haemoglobin; KNH: Kenyatta National Hospital; Type I DM: Type 1 diabetes mellitus; Type II DM: Type 2 diabetes mellitus.

## Competing interests

The authors declare that they have no competing interests.

## Authors’ contributions

DMM was responsible for conception, design, data collection, analysis, interpretation and write up of the manuscript; JMK involved in design, data collection, drafting and revision of manuscript; CMM involved in data collection, analysis, and drafting of manuscript; PWM involved in conception, design and critical revision of manuscript. All authors read and approved the final version of the manuscript.
